# Methyl 3′-benzyl-4′-(2,4-di­chloro­phen­yl)-1′-methyl-2-oxo-1-propyl­spiro­[indoline-3,2′-pyrrolidine]-3′-carboxy­l­ate

**DOI:** 10.1107/S1600536814002621

**Published:** 2014-02-12

**Authors:** S. Karthikeyan, K. Sethusankar, Anthonisamy Devaraj, Manickam Bakthadoss

**Affiliations:** a Department of Physics, RKM Vivekananda College (Autonomous), Chennai 600 004, India; b Department of Organic Chemistry, University of Madras, Maraimalai Campus, Chennai 600 025, India

## Abstract

In the title compound, C_30_H_30_Cl_2_N_2_O_3_, the indole ring system is roughly planar, with a maximum deviation of 0.1039 (18) Å for the carbonyl C atom, and makes a dihedral angle of 86.61 (9)° with the mean plane of the pyrrolidine ring. This spiro pyrrolidine ring adopts an envelope conformation with the N atom at the flap position. The pyrrole ring of the indole ring system adopts a twisted conformation on the C—C(=O) bond. The mol­ecular structure is stabilized by an intra­molecular C—H⋯O hydrogen bond, which generates an *S*(6) ring motif, and a π–π inter­action [centroid–centroid distance = 3.6577 (12) Å] involving the 2,4-di­chloro­phenyl ring and the benzyl ring. In the crystal, mol­ecules are linked *via* C—H⋯O hydrogen bonds, forming *C*(9) chains running parallel to [10-1].

## Related literature   

For the biological activity of spiro-pyrrolidine and oxindole derivatives, see: Peddi *et al.* (2004 ▶); Rajeswaran *et al.* (1999 ▶). For a related crystal structure, see: Jagadeesan *et al.* (2013 ▶). For graph-set motif notation, see: Bernstein *et al.* (1995 ▶). For ring puckering analysis, see: Cremer & Pople (1975 ▶). For bond-length distortions in small rings, see: Allen (1981 ▶).
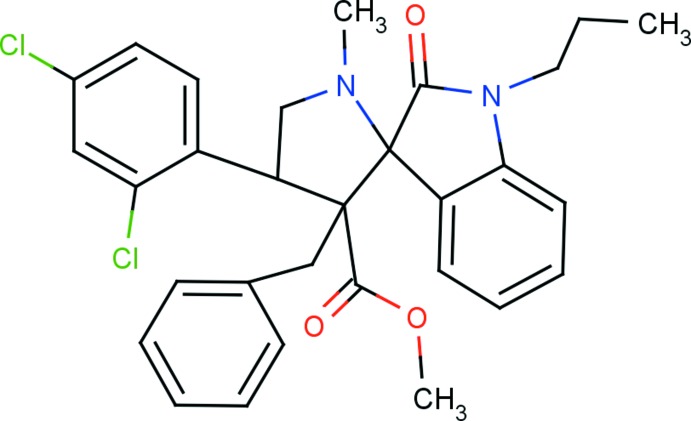


## Experimental   

### 

#### Crystal data  

C_30_H_30_Cl_2_N_2_O_3_
*M* *_r_* = 537.46
Monoclinic, 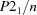

*a* = 7.5563 (4) Å
*b* = 28.8497 (16) Å
*c* = 12.4274 (8) Å
β = 90.433 (3)°
*V* = 2709.1 (3) Å^3^
*Z* = 4
Mo *K*α radiation
μ = 0.27 mm^−1^
*T* = 293 K
0.35 × 0.30 × 0.25 mm

#### Data collection  

Bruker Kappa APEXII CCD diffractometer
31802 measured reflections
7794 independent reflections
4985 reflections with *I* > 2σ(*I*)
*R*_int_ = 0.029

#### Refinement  

*R*[*F* ^2^ > 2σ(*F* ^2^)] = 0.051
*wR*(*F* ^2^) = 0.167
*S* = 1.00
7794 reflections
337 parameters
H-atom parameters constrained
Δρ_max_ = 0.43 e Å^−3^
Δρ_min_ = −0.58 e Å^−3^

### 

Data collection: *APEX2* (Bruker, 2008 ▶); cell refinement: *SAINT* (Bruker, 2008 ▶); data reduction: *SAINT*; program(s) used to solve structure: *SHELXS97* (Sheldrick, 2008 ▶); program(s) used to refine structure: *SHELXL97* (Sheldrick, 2008 ▶); molecular graphics: *ORTEP-3 for Windows* (Farrugia, 2012 ▶) and *Mercury* (Macrae *et al.*, 2008 ▶); software used to prepare material for publication: *SHELXL97* and *PLATON* (Spek, 2009 ▶).

## Supplementary Material

Crystal structure: contains datablock(s) global, I. DOI: 10.1107/S1600536814002621/su2694sup1.cif

Structure factors: contains datablock(s) I. DOI: 10.1107/S1600536814002621/su2694Isup2.hkl

Supporting information file. DOI: 10.1107/S1600536814002621/su2694Isup3.cml

CCDC reference:

Additional supporting information:  crystallographic information; 3D view; checkCIF report

## Figures and Tables

**Table 1:** Hydrogen-bond geometry (Å, °)

*D*—H⋯*A*	*D*—H	H⋯*A*	*D*⋯*A*	*D*—H⋯*A*
C18—H18*B*⋯O1	0.97	2.39	3.073 (2)	127
C13—H13⋯O2^i^	0.93	2.46	3.132 (2)	129

Symmetry code: (i) 
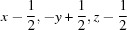
.

**Fig. 1.**
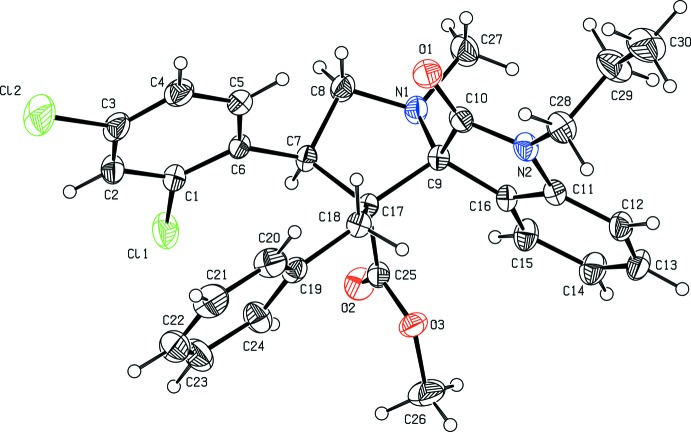
The molecular structure of the title molecule, with atom labelling. Displacement ellipsoids are drawn at 30% probability level.

**Fig. 2.**
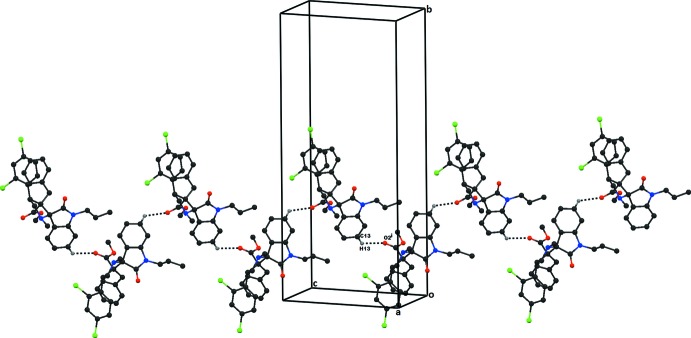
The crystal packing of the title compound viewed along the a axis, showing the formation of infinite C(9) chains. Dashed lines indicate C—H···O hydrogen bonds - see Table 1 for details.

## supplementary crystallographic information

### 1. Comment

Spiro-pyrrolidine derivatives are unique tetracyclic 5-HT(2 A) receptor antagonists (Peddi *et al.*, 2004). Oxindole derivatives help to treat and prevent diabetic complications arising from elevated levels of sorbitol and act as aldose reductase inhibitor (Rajeswaran *et al.*, 1999).

The molecular structure of the title compound is illustrated in Fig 1. In the molecule, there is C-H···O hydrogen bond, forming an *S*(6) ring motif (Bernstein *et al.*, 1995), and a π–π interaction present [*Cg*(1)···*Cg*(2) = 3.6577 (12) Å, where Cg1 and Cg2 are the centroids of rings C1-C6 and C19-C24, respectively]. The indole ring system is essentially planar with a maximum deviation of 0.1039 (18) Å for atom C10. The mean plane of the indole ring system forms a dihedral angle of 86.61 (9) ° with the mean plane of the pyrrolidine five membered ring. The latter forms a dihedral angle of 80.80 (11) ° with the benzyl ring which shows that they are almost orthogonal. Atom O1 deviates from the mean plane of the indole ring system by 0.2776 (15) Å. The molecular dimensions in the title compound are in excellent agreement with the corresponding dimensions reported in another closely related compound (Jagadeesan *et al.*, 2013).

The spiro-pyrrolidine ring adopts an envelope conformation with atom N1 at the flap position. The distance to the flap position from the mean plane of the spiro carbon is 0.2524 (17) Å; the puckering parameters (Cremer & Pople, 1975) of the ring are Q_2_ = 0.4033 (18) Å and φ_2_ = 8.5 (3)°. The pyrrole ring system adopts a twisted conformation on bond C9-C10 with the deviation of -0.0559 (18) Å. The central spiro-pyrrolidine ring is perpendicular to the dichloro phenyl ring with a dihedral angle of 87.33 (10)°. The carbonyl group and the benzyl ring have a (+)anti-clinal conformation with torsion angle C18—C17—C25—O2 = 148.57 (17)°.

In the benzene ring (C11—C16) of the indole ring system, the expansion of the ipso angles at C11, C13 and C14 [121.37 (16), 121.56 (18) and 120.37 (18)°, respectively] and contraction of the apical angles at C12, C15 and C16 [117.81 (18), 118.82 (18) and 119.97 (18)°, respectively] are caused by the fusion of the smaller pyrrole ring to the six-membered benzene ring and the strain is taken up by the angular distortion rather than by bond-length distortions (Allen, 1981). The carboxyl group and oxindole ring system are (-)*syn*-clinal to each other with the torsion angle C9—C17—C25—O2 = -89.82 (19)°.

The crystal packing (Fig. 2 and Table 1) is stabilized by a C-H···O hydrogen bond resulting in the formation of *C*(9) chains running parallel to [1 0 -1].

### 2. Experimental

A mixture of (*E*)-methyl 2-benzyl-3-(2,4-dichlorophenyl)acrylate (2 mmol), *N*-propyl isatin (2 mmol) and sarcosine (2 mmol) in acetonitrile (8 ml) was refluxed for 12 h. After the completion of the reaction as indicated by TLC, the reaction mixture was concentrated. The resulting crude mass was diluted with water (10 ml) and extracted with ethyl acetate (3 × 10 ml). The combined organic layers were washed with brine (2 × 10 ml) and dried over anhydrous Na_2_SO_4_. The organic layer was concentrated and the residue purified by column chromatography on silica gel (Acme 100–200 mesh), using ethyl acetate:hexanes (2:8) to afford the title compound as a colourless solid in (62%) yield. Block-like colourless crystals were obtained by slow evaporation of a solution in CHCl_3_.

### 3. Refinement

The H atoms could all be localed in difference electron-density maps. In the final cycles of refinement they were treated as riding atoms and their distances were geometrically constrained: C-H = 0.93 and 0.96 Å for CH and CH_3_ H atoms, respectively, with *U*_iso_(H) = 1.5 *U*_eq_(C-methyl) and = 1.2*U*_eq_(C) for other H atoms.

### Crystal data

**Table:**

C_30_H_30_Cl_2_N_2_O_3_	*F*(000) = 1128
*M**_r_* = 537.46	*D*_x_ = 1.318 Mg m^−^^3^
Monoclinic, *P*2_1_/*n*	Mo *K*α radiation, λ = 0.71073 Å
Hall symbol: -P 2yn	Cell parameters from 7794 reflections
*a* = 7.5563 (4) Å	θ = 2.2–29.9°
*b* = 28.8497 (16) Å	µ = 0.27 mm^−^^1^
*c* = 12.4274 (8) Å	*T* = 293 K
β = 90.433 (3)°	Block, colourless
*V* = 2709.1 (3) Å^3^	0.35 × 0.30 × 0.25 mm
*Z* = 4	

### Data collection

**Table:**

Bruker Kappa APEXII CCD diffractometer	4985 reflections with *I* > 2σ(*I*)
Radiation source: fine-focus sealed tube	*R*_int_ = 0.029
Graphite monochromator	θ_max_ = 29.9°, θ_min_ = 2.2°
ω scans	*h* = −10→7
31802 measured reflections	*k* = −37→40
7794 independent reflections	*l* = −17→16

### Refinement

**Table:**

Refinement on *F*^2^	Primary atom site location: structure-invariant direct methods
Least-squares matrix: full	Secondary atom site location: difference Fourier map
*R*[*F*^2^ > 2σ(*F*^2^)] = 0.051	Hydrogen site location: inferred from neighbouring sites
*wR*(*F*^2^) = 0.167	H-atom parameters constrained
*S* = 1.00	*w* = 1/[σ^2^(*F*_o_^2^) + (0.0918*P*)^2^ + 0.3965*P*] where *P* = (*F*_o_^2^ + 2*F*_c_^2^)/3
7794 reflections	(Δ/σ)_max_ < 0.001
337 parameters	Δρ_max_ = 0.43 e Å^−^^3^
0 restraints	Δρ_min_ = −0.58 e Å^−^^3^

### Special details

**Table:**

Geometry. All e.s.d.'s (except the e.s.d. in the dihedral angle between two l.s. planes) are estimated using the full covariance matrix. The cell e.s.d.'s are taken into account individually in the estimation of e.s.d.'s in distances, angles and torsion angles; correlations between e.s.d.'s in cell parameters are only used when they are defined by crystal symmetry. An approximate (isotropic) treatment of cell e.s.d.'s is used for estimating e.s.d.'s involving l.s. planes.
Refinement. Refinement of *F*^2^ against ALL reflections. The weighted *R*-factor *wR* and goodness of fit *S* are based on *F*^2^, conventional *R*-factors *R* are based on *F*, with *F* set to zero for negative *F*^2^. The threshold expression of *F*^2^ > σ(*F*^2^) is used only for calculating *R*-factors(gt) *etc*. and is not relevant to the choice of reflections for refinement. *R*-factors based on *F*^2^ are statistically about twice as large as those based on *F*, and *R*- factors based on ALL data will be even larger.

### Fractional atomic coordinates and isotropic or equivalent isotropic displacement parameters (Å^2^)

**Table:**

	*x*	*y*	*z*	*U*_iso_*/*U*_eq_	
C1	0.3859 (2)	0.04008 (6)	0.32495 (15)	0.0424 (4)	
C2	0.3770 (3)	−0.00713 (7)	0.34519 (18)	0.0512 (5)	
H2	0.3748	−0.0183	0.4153	0.061*	
C3	0.3714 (2)	−0.03682 (7)	0.25925 (19)	0.0520 (5)	
C4	0.3773 (3)	−0.02096 (7)	0.15521 (18)	0.0509 (5)	
H4	0.3762	−0.0416	0.0977	0.061*	
C5	0.3851 (2)	0.02637 (6)	0.13764 (16)	0.0441 (4)	
H5	0.3897	0.0373	0.0673	0.053*	
C6	0.3861 (2)	0.05816 (6)	0.22188 (14)	0.0361 (4)	
C7	0.3906 (2)	0.10974 (6)	0.20185 (14)	0.0349 (3)	
H7	0.4141	0.1247	0.2713	0.042*	
C8	0.5393 (2)	0.12406 (6)	0.12614 (17)	0.0456 (4)	
H8A	0.6510	0.1268	0.1645	0.055*	
H8B	0.5524	0.1019	0.0680	0.055*	
C9	0.2936 (2)	0.16388 (5)	0.05789 (13)	0.0330 (3)	
C10	0.2552 (2)	0.13941 (6)	−0.05080 (14)	0.0385 (4)	
C11	0.1097 (2)	0.20864 (6)	−0.05612 (13)	0.0358 (4)	
C12	0.0117 (2)	0.24610 (7)	−0.09082 (16)	0.0455 (4)	
H12	−0.0537	0.2448	−0.1545	0.055*	
C13	0.0137 (3)	0.28541 (7)	−0.02830 (18)	0.0519 (5)	
H13	−0.0537	0.3109	−0.0494	0.062*	
C14	0.1123 (3)	0.28797 (7)	0.06395 (18)	0.0530 (5)	
H14	0.1141	0.3153	0.1036	0.064*	
C15	0.2100 (3)	0.24996 (6)	0.09897 (16)	0.0454 (4)	
H15	0.2778	0.2516	0.1616	0.054*	
C16	0.2047 (2)	0.20999 (5)	0.03956 (13)	0.0342 (3)	
C17	0.21755 (19)	0.13252 (5)	0.15373 (12)	0.0298 (3)	
C18	0.0724 (2)	0.09876 (6)	0.11544 (14)	0.0358 (4)	
H18A	−0.0209	0.1169	0.0820	0.043*	
H18B	0.1227	0.0793	0.0598	0.043*	
C19	−0.0128 (2)	0.06708 (6)	0.19792 (15)	0.0387 (4)	
C20	−0.0855 (3)	0.02613 (7)	0.1602 (2)	0.0550 (5)	
H20	−0.0829	0.0197	0.0869	0.066*	
C21	−0.1623 (3)	−0.00549 (8)	0.2298 (3)	0.0697 (7)	
H21	−0.2107	−0.0328	0.2028	0.084*	
C22	−0.1672 (3)	0.00321 (9)	0.3375 (2)	0.0694 (7)	
H22	−0.2169	−0.0183	0.3842	0.083*	
C23	−0.0990 (3)	0.04354 (9)	0.3761 (2)	0.0653 (6)	
H23	−0.1033	0.0498	0.4494	0.078*	
C24	−0.0228 (3)	0.07536 (7)	0.30671 (17)	0.0517 (5)	
H24	0.0225	0.1029	0.3344	0.062*	
C25	0.1402 (2)	0.16665 (6)	0.23502 (13)	0.0357 (4)	
C26	−0.1178 (4)	0.20778 (9)	0.2834 (2)	0.0752 (7)	
H26A	−0.1124	0.1962	0.3558	0.113*	
H26B	−0.2392	0.2110	0.2614	0.113*	
H26C	−0.0605	0.2374	0.2802	0.113*	
C27	0.5916 (3)	0.18816 (9)	0.0023 (2)	0.0626 (6)	
H27A	0.7088	0.1930	0.0305	0.094*	
H27B	0.5430	0.2172	−0.0214	0.094*	
H27C	0.5966	0.1671	−0.0574	0.094*	
C28	0.0617 (3)	0.15200 (8)	−0.21054 (15)	0.0505 (5)	
H28A	−0.0588	0.1634	−0.2150	0.061*	
H28B	0.0571	0.1184	−0.2136	0.061*	
C29	0.1629 (3)	0.16950 (10)	−0.30642 (18)	0.0690 (7)	
H29A	0.1616	0.2031	−0.3065	0.083*	
H29B	0.2852	0.1595	−0.3007	0.083*	
C30	0.0850 (4)	0.15207 (12)	−0.41017 (19)	0.0840 (8)	
H30A	0.0914	0.1188	−0.4118	0.126*	
H30B	0.1501	0.1646	−0.4695	0.126*	
H30C	−0.0365	0.1616	−0.4156	0.126*	
N1	0.48008 (18)	0.16871 (5)	0.08585 (13)	0.0412 (3)	
N2	0.1382 (2)	0.16591 (5)	−0.10730 (11)	0.0412 (3)	
O1	0.3160 (2)	0.10260 (5)	−0.08116 (11)	0.0526 (3)	
O2	0.21833 (19)	0.18395 (5)	0.30852 (11)	0.0523 (3)	
O3	−0.02944 (16)	0.17580 (4)	0.21260 (10)	0.0455 (3)	
Cl1	0.39756 (9)	0.07585 (2)	0.43710 (4)	0.06494 (18)	
Cl2	0.36248 (10)	−0.09601 (2)	0.28361 (7)	0.0864 (2)	

### Atomic displacement parameters (Å^2^)

**Table:**

	*U*^11^	*U*^22^	*U*^33^	*U*^12^	*U*^13^	*U*^23^
C1	0.0429 (9)	0.0406 (9)	0.0438 (10)	0.0073 (7)	0.0002 (7)	0.0091 (8)
C2	0.0486 (11)	0.0475 (11)	0.0574 (12)	0.0055 (8)	0.0043 (9)	0.0206 (9)
C3	0.0426 (10)	0.0336 (9)	0.0799 (15)	0.0030 (7)	0.0064 (9)	0.0123 (9)
C4	0.0512 (11)	0.0383 (10)	0.0631 (13)	0.0060 (8)	0.0058 (9)	−0.0036 (9)
C5	0.0458 (10)	0.0399 (10)	0.0467 (10)	0.0081 (8)	0.0065 (8)	0.0042 (8)
C6	0.0334 (8)	0.0331 (8)	0.0419 (9)	0.0061 (6)	0.0006 (7)	0.0072 (7)
C7	0.0353 (8)	0.0329 (8)	0.0363 (9)	0.0024 (6)	−0.0057 (6)	0.0058 (6)
C8	0.0335 (9)	0.0448 (10)	0.0586 (12)	0.0027 (7)	−0.0015 (8)	0.0143 (8)
C9	0.0354 (8)	0.0310 (8)	0.0324 (8)	0.0005 (6)	−0.0035 (6)	0.0026 (6)
C10	0.0463 (9)	0.0366 (9)	0.0328 (9)	−0.0014 (7)	0.0035 (7)	0.0020 (7)
C11	0.0383 (8)	0.0363 (9)	0.0328 (8)	−0.0024 (7)	−0.0006 (6)	0.0044 (6)
C12	0.0428 (9)	0.0507 (11)	0.0429 (10)	0.0034 (8)	−0.0064 (8)	0.0115 (8)
C13	0.0530 (11)	0.0409 (10)	0.0617 (13)	0.0120 (8)	−0.0024 (9)	0.0129 (9)
C14	0.0661 (13)	0.0320 (9)	0.0607 (13)	0.0036 (9)	−0.0033 (10)	−0.0016 (8)
C15	0.0566 (11)	0.0331 (9)	0.0462 (10)	−0.0008 (8)	−0.0112 (8)	0.0003 (7)
C16	0.0383 (8)	0.0300 (8)	0.0344 (8)	−0.0008 (6)	−0.0033 (6)	0.0054 (6)
C17	0.0314 (7)	0.0287 (7)	0.0292 (8)	0.0016 (6)	−0.0038 (6)	0.0016 (6)
C18	0.0356 (8)	0.0346 (8)	0.0373 (9)	−0.0021 (6)	−0.0042 (7)	−0.0016 (7)
C19	0.0296 (8)	0.0363 (9)	0.0500 (10)	0.0000 (6)	−0.0019 (7)	0.0034 (7)
C20	0.0485 (11)	0.0447 (11)	0.0719 (14)	−0.0079 (8)	0.0065 (10)	−0.0063 (10)
C21	0.0538 (12)	0.0422 (12)	0.113 (2)	−0.0126 (9)	0.0122 (13)	0.0043 (13)
C22	0.0523 (12)	0.0620 (14)	0.094 (2)	−0.0061 (11)	0.0183 (12)	0.0274 (13)
C23	0.0617 (13)	0.0741 (16)	0.0603 (14)	−0.0106 (11)	0.0110 (11)	0.0193 (12)
C24	0.0517 (11)	0.0534 (12)	0.0499 (12)	−0.0129 (9)	0.0014 (9)	0.0072 (9)
C25	0.0415 (9)	0.0326 (8)	0.0329 (8)	0.0021 (7)	−0.0021 (7)	0.0020 (6)
C26	0.0779 (16)	0.0790 (17)	0.0689 (16)	0.0340 (13)	0.0173 (12)	−0.0145 (13)
C27	0.0469 (11)	0.0669 (14)	0.0742 (15)	−0.0074 (10)	0.0080 (10)	0.0274 (12)
C28	0.0590 (11)	0.0574 (12)	0.0349 (10)	−0.0063 (9)	−0.0075 (8)	−0.0045 (8)
C29	0.0680 (14)	0.101 (2)	0.0381 (11)	−0.0087 (13)	0.0017 (10)	0.0019 (11)
C30	0.0926 (19)	0.124 (3)	0.0351 (12)	0.0123 (17)	0.0002 (12)	−0.0111 (13)
N1	0.0341 (7)	0.0395 (8)	0.0498 (9)	−0.0018 (6)	−0.0025 (6)	0.0130 (6)
N2	0.0517 (8)	0.0428 (8)	0.0289 (7)	0.0006 (7)	−0.0058 (6)	−0.0009 (6)
O1	0.0706 (9)	0.0416 (7)	0.0459 (8)	0.0095 (6)	0.0077 (6)	−0.0063 (6)
O2	0.0649 (8)	0.0495 (8)	0.0424 (8)	0.0023 (6)	−0.0125 (6)	−0.0131 (6)
O3	0.0418 (7)	0.0473 (7)	0.0473 (7)	0.0131 (5)	0.0022 (5)	−0.0050 (6)
Cl1	0.0957 (4)	0.0589 (3)	0.0401 (3)	0.0111 (3)	−0.0062 (3)	0.0068 (2)
Cl2	0.0968 (5)	0.0375 (3)	0.1251 (6)	−0.0044 (3)	0.0081 (4)	0.0214 (3)

### Geometric parameters (Å, º)

**Table:**

C1—C6	1.383 (2)	C17—C18	1.539 (2)
C1—C2	1.387 (3)	C18—C19	1.520 (2)
C1—Cl1	1.736 (2)	C18—H18A	0.9700
C2—C3	1.369 (3)	C18—H18B	0.9700
C2—H2	0.9300	C19—C24	1.376 (3)
C3—C4	1.373 (3)	C19—C20	1.384 (3)
C3—Cl2	1.7356 (19)	C20—C21	1.388 (3)
C4—C5	1.384 (3)	C20—H20	0.9300
C4—H4	0.9300	C21—C22	1.362 (4)
C5—C6	1.392 (3)	C21—H21	0.9300
C5—H5	0.9300	C22—C23	1.359 (4)
C6—C7	1.509 (2)	C22—H22	0.9300
C7—C8	1.527 (2)	C23—C24	1.388 (3)
C7—C17	1.577 (2)	C23—H23	0.9300
C7—H7	0.9800	C24—H24	0.9300
C8—N1	1.451 (2)	C25—O2	1.193 (2)
C8—H8A	0.9700	C25—O3	1.336 (2)
C8—H8B	0.9700	C26—O3	1.442 (2)
C9—N1	1.456 (2)	C26—H26A	0.9600
C9—C16	1.507 (2)	C26—H26B	0.9600
C9—C10	1.550 (2)	C26—H26C	0.9600
C9—C17	1.605 (2)	C27—N1	1.455 (2)
C10—O1	1.218 (2)	C27—H27A	0.9600
C10—N2	1.360 (2)	C27—H27B	0.9600
C11—C12	1.378 (2)	C27—H27C	0.9600
C11—C16	1.385 (2)	C28—N2	1.460 (2)
C11—N2	1.404 (2)	C28—C29	1.508 (3)
C12—C13	1.375 (3)	C28—H28A	0.9700
C12—H12	0.9300	C28—H28B	0.9700
C13—C14	1.364 (3)	C29—C30	1.500 (3)
C13—H13	0.9300	C29—H29A	0.9700
C14—C15	1.390 (3)	C29—H29B	0.9700
C14—H14	0.9300	C30—H30A	0.9600
C15—C16	1.370 (2)	C30—H30B	0.9600
C15—H15	0.9300	C30—H30C	0.9600
C17—C25	1.530 (2)		
			
C6—C1—C2	122.61 (19)	C19—C18—H18A	107.7
C6—C1—Cl1	121.26 (14)	C17—C18—H18A	107.7
C2—C1—Cl1	116.14 (15)	C19—C18—H18B	107.7
C3—C2—C1	118.30 (18)	C17—C18—H18B	107.7
C3—C2—H2	120.9	H18A—C18—H18B	107.1
C1—C2—H2	120.9	C24—C19—C20	117.20 (18)
C2—C3—C4	121.67 (18)	C24—C19—C18	125.81 (16)
C2—C3—Cl2	118.71 (17)	C20—C19—C18	116.99 (17)
C4—C3—Cl2	119.60 (18)	C19—C20—C21	121.1 (2)
C3—C4—C5	118.64 (19)	C19—C20—H20	119.4
C3—C4—H4	120.7	C21—C20—H20	119.4
C5—C4—H4	120.7	C22—C21—C20	120.4 (2)
C4—C5—C6	122.10 (18)	C22—C21—H21	119.8
C4—C5—H5	118.9	C20—C21—H21	119.8
C6—C5—H5	118.9	C23—C22—C21	119.5 (2)
C1—C6—C5	116.61 (16)	C23—C22—H22	120.3
C1—C6—C7	121.66 (16)	C21—C22—H22	120.3
C5—C6—C7	121.72 (16)	C22—C23—C24	120.3 (2)
C6—C7—C8	112.70 (14)	C22—C23—H23	119.8
C6—C7—C17	116.98 (13)	C24—C23—H23	119.8
C8—C7—C17	105.37 (13)	C19—C24—C23	121.5 (2)
C6—C7—H7	107.1	C19—C24—H24	119.3
C8—C7—H7	107.1	C23—C24—H24	119.3
C17—C7—H7	107.1	O2—C25—O3	123.05 (16)
N1—C8—C7	103.09 (14)	O2—C25—C17	125.83 (15)
N1—C8—H8A	111.1	O3—C25—C17	111.11 (14)
C7—C8—H8A	111.1	O3—C26—H26A	109.5
N1—C8—H8B	111.1	O3—C26—H26B	109.5
C7—C8—H8B	111.1	H26A—C26—H26B	109.5
H8A—C8—H8B	109.1	O3—C26—H26C	109.5
N1—C9—C16	112.40 (13)	H26A—C26—H26C	109.5
N1—C9—C10	115.24 (14)	H26B—C26—H26C	109.5
C16—C9—C10	100.96 (13)	N1—C27—H27A	109.5
N1—C9—C17	103.19 (12)	N1—C27—H27B	109.5
C16—C9—C17	116.61 (13)	H27A—C27—H27B	109.5
C10—C9—C17	108.92 (12)	N1—C27—H27C	109.5
O1—C10—N2	125.15 (17)	H27A—C27—H27C	109.5
O1—C10—C9	126.75 (16)	H27B—C27—H27C	109.5
N2—C10—C9	108.09 (14)	N2—C28—C29	113.74 (17)
C12—C11—C16	121.37 (16)	N2—C28—H28A	108.8
C12—C11—N2	129.12 (16)	C29—C28—H28A	108.8
C16—C11—N2	109.46 (14)	N2—C28—H28B	108.8
C13—C12—C11	117.81 (18)	C29—C28—H28B	108.8
C13—C12—H12	121.1	H28A—C28—H28B	107.7
C11—C12—H12	121.1	C30—C29—C28	111.6 (2)
C14—C13—C12	121.56 (18)	C30—C29—H29A	109.3
C14—C13—H13	119.2	C28—C29—H29A	109.3
C12—C13—H13	119.2	C30—C29—H29B	109.3
C13—C14—C15	120.37 (18)	C28—C29—H29B	109.3
C13—C14—H14	119.8	H29A—C29—H29B	108.0
C15—C14—H14	119.8	C29—C30—H30A	109.5
C16—C15—C14	118.82 (18)	C29—C30—H30B	109.5
C16—C15—H15	120.6	H30A—C30—H30B	109.5
C14—C15—H15	120.6	C29—C30—H30C	109.5
C15—C16—C11	119.97 (15)	H30A—C30—H30C	109.5
C15—C16—C9	130.58 (16)	H30B—C30—H30C	109.5
C11—C16—C9	109.42 (14)	C8—N1—C27	114.20 (15)
C25—C17—C18	109.67 (13)	C8—N1—C9	107.01 (13)
C25—C17—C7	109.73 (13)	C27—N1—C9	115.56 (15)
C18—C17—C7	116.11 (13)	C10—N2—C11	111.17 (14)
C25—C17—C9	105.54 (12)	C10—N2—C28	123.42 (16)
C18—C17—C9	112.66 (13)	C11—N2—C28	125.32 (15)
C7—C17—C9	102.46 (11)	C25—O3—C26	116.54 (17)
C19—C18—C17	118.45 (14)		
			
C6—C1—C2—C3	1.2 (3)	C16—C9—C17—C25	−28.00 (17)
Cl1—C1—C2—C3	−178.64 (15)	C10—C9—C17—C25	−141.37 (13)
C1—C2—C3—C4	1.1 (3)	N1—C9—C17—C18	−144.62 (13)
C1—C2—C3—Cl2	179.34 (14)	C16—C9—C17—C18	91.65 (16)
C2—C3—C4—C5	−1.5 (3)	C10—C9—C17—C18	−21.72 (17)
Cl2—C3—C4—C5	−179.75 (15)	N1—C9—C17—C7	−19.13 (15)
C3—C4—C5—C6	−0.3 (3)	C16—C9—C17—C7	−142.86 (14)
C2—C1—C6—C5	−2.9 (3)	C10—C9—C17—C7	103.78 (14)
Cl1—C1—C6—C5	176.96 (13)	C25—C17—C18—C19	−62.32 (18)
C2—C1—C6—C7	178.20 (16)	C7—C17—C18—C19	62.74 (19)
Cl1—C1—C6—C7	−2.0 (2)	C9—C17—C18—C19	−179.56 (13)
C4—C5—C6—C1	2.4 (3)	C17—C18—C19—C24	24.6 (3)
C4—C5—C6—C7	−178.66 (16)	C17—C18—C19—C20	−154.86 (16)
C1—C6—C7—C8	127.31 (18)	C24—C19—C20—C21	−1.1 (3)
C5—C6—C7—C8	−51.6 (2)	C18—C19—C20—C21	178.42 (18)
C1—C6—C7—C17	−110.32 (18)	C19—C20—C21—C22	−0.1 (4)
C5—C6—C7—C17	70.8 (2)	C20—C21—C22—C23	1.1 (4)
C6—C7—C8—N1	158.73 (15)	C21—C22—C23—C24	−0.8 (4)
C17—C7—C8—N1	30.04 (18)	C20—C19—C24—C23	1.4 (3)
N1—C9—C10—O1	50.1 (2)	C18—C19—C24—C23	−178.06 (18)
C16—C9—C10—O1	171.47 (17)	C22—C23—C24—C19	−0.5 (4)
C17—C9—C10—O1	−65.2 (2)	C18—C17—C25—O2	148.57 (17)
N1—C9—C10—N2	−130.73 (15)	C7—C17—C25—O2	19.9 (2)
C16—C9—C10—N2	−9.37 (17)	C9—C17—C25—O2	−89.82 (19)
C17—C9—C10—N2	113.91 (14)	C18—C17—C25—O3	−32.52 (18)
C16—C11—C12—C13	1.2 (3)	C7—C17—C25—O3	−161.18 (13)
N2—C11—C12—C13	−175.90 (17)	C9—C17—C25—O3	89.09 (15)
C11—C12—C13—C14	1.5 (3)	N2—C28—C29—C30	176.8 (2)
C12—C13—C14—C15	−1.9 (3)	C7—C8—N1—C27	−174.07 (17)
C13—C14—C15—C16	−0.3 (3)	C7—C8—N1—C9	−44.87 (18)
C14—C15—C16—C11	2.9 (3)	C16—C9—N1—C8	166.61 (15)
C14—C15—C16—C9	−179.25 (17)	C10—C9—N1—C8	−78.45 (18)
C12—C11—C16—C15	−3.5 (3)	C17—C9—N1—C8	40.15 (17)
N2—C11—C16—C15	174.18 (16)	C16—C9—N1—C27	−65.0 (2)
C12—C11—C16—C9	178.32 (15)	C10—C9—N1—C27	50.0 (2)
N2—C11—C16—C9	−4.06 (18)	C17—C9—N1—C27	168.57 (16)
N1—C9—C16—C15	−46.7 (2)	O1—C10—N2—C11	−173.11 (16)
C10—C9—C16—C15	−170.01 (18)	C9—C10—N2—C11	7.72 (19)
C17—C9—C16—C15	72.2 (2)	O1—C10—N2—C28	3.8 (3)
N1—C9—C16—C11	131.31 (15)	C9—C10—N2—C28	−175.39 (15)
C10—C9—C16—C11	7.97 (16)	C12—C11—N2—C10	174.91 (17)
C17—C9—C16—C11	−109.83 (16)	C16—C11—N2—C10	−2.48 (19)
C6—C7—C17—C25	115.66 (16)	C12—C11—N2—C28	−1.9 (3)
C8—C7—C17—C25	−118.25 (15)	C16—C11—N2—C28	−179.30 (16)
C6—C7—C17—C18	−9.4 (2)	C29—C28—N2—C10	−93.2 (2)
C8—C7—C17—C18	116.72 (16)	C29—C28—N2—C11	83.2 (2)
C6—C7—C17—C9	−132.57 (14)	O2—C25—O3—C26	−0.9 (3)
C8—C7—C17—C9	−6.48 (17)	C17—C25—O3—C26	−179.80 (17)
N1—C9—C17—C25	95.73 (14)		

### Hydrogen-bond geometry (Å, º)

**Table:**

*D*—H···*A*	*D*—H	H···*A*	*D*···*A*	*D*—H···*A*
C18—H18*B*···O1	0.97	2.39	3.073 (2)	127
C13—H13···O2^i^	0.93	2.46	3.132 (2)	129

Symmetry code: (i) *x*−1/2, −*y*+1/2, *z*−1/2.
